# Book Notices

**Published:** 1878-01-20

**Authors:** 


### BOOK NOTICES.

Modern Medical Therapeutics; a Compendium of recent formulæ and Specific Therapeutical Directions, from the Practice of Eminent Contemporary Physicians, American and Foreign. By George H. Naphey's, A.M., M.D., etc. Fifth edition, enlarged and revised 1 vol. 8.vo. 600 pages. Price $4.00—full leather $5.00. D. G. Brinton, Philadelphia.

Only a few months ago we had occasion to notice the fourth edition of this excellent work. That a fifth and more enlarged edition should be so soon called for, evinces the approval of the work by the profession, and its happy adoptation to the wants of the practitioner. The present volume, containing many valuable additions and improvements, will doubtless be equally sought after. It will be found a most valuable aid and convenient book of reference for the daily practitioner.

Modern Surgical Thrapeutics; A Compendium of Current Formulæ, Approved Dressings, and Specific Methods for the Treatment of Surgical Diseases and Injuries—a Companion Volume to the work on Medical Therapeutics. Price, $4.00 Full leather, $5.00. D. G. Brinton, Publisher—Philadelphia.

This work meets a want greatly felt, not only by the surgeon proper, but by every practitioner—as every one must possess some knowledge of surgical practice. In presenting this volume to the public the editor remarks that he can claim for it that it fills a place in medical literature now entirely unoccupied. Its chief purpose is to set forth the medical aspects of surgery. There seems to be no work extant which contains the therapeutics of surgery strictly, with the formulæ and practice and systematic directions of distinguished surgeons, and a specific treatment of surgical diseases and injuries. We can safely recommend this work to our professional readers as eminently practical.

A Guide to Therapeutics and Materia Medica; By Robert Farquharson, M.D., Edinburg, F. R. C. P., London, Lecturer on Materia Medica, etc. Enlarged and adopted to the United States Pharmacopœa, by Frank Woodbury, M.D., Member of the Academy of Natural Sciences, Phil., etc. Henry C. Lea, Publisher, Philadelphia.

This is a compact volume of 400 pages, containing much practical and important information. The corresponding effect of drugs upon both the healthy and diseased system are presented in parallel columns. Copious notes are also given embodying the latest revision of the United States Pharmacopœa, together with antidotes to poisons, and the more important of the later remedial agents of the day. The work cannot fail to interest and profit both the student and practitioner.

Catalogue No. VI., of Western Electric Manufacturing Company, Electro-Medical and Surgical Apparatus, Electricity and Surgery, 220 Kinzie st., Chicago.

A very neatly bound and elegantly gotten up pamphlet of 77 pages, containing cuts and illustrations of various batteries and electrical apparatus, with instructions as to use, therapeutic and physiological effects, etc. Medical men who desire to post themselves in this important department would do well to examine this work.

An Address Introductory to the 12th Course of Lectures in the Medical Department of the University of Philadelphia, Delivered October 1, 1877, on Higher Medical Education. By William Pepper, A.M. M.D., Prof. of Clinical Medicine.

This address is highly interesting, and should be read by every member of the profession. We propose to notice it more fully at another time.

A New Medical Association in Atlanta.—At a preliminary meeting, recently held by a respectable number of medical gentlemen of the city, the initial steps were taken for the organization of a medical association, having in view the advancement of medical science, and the social and professional good of its members. The first meeting will be held at the office of Dr. J. J. Knott, on Friday evening, February 1, when the election of officers will take place, and a permanent organization effected. A brief of cases reported may be expected in future issues of the Record.

Receipted for 1878.—Drs P W Callihan, A T Park, M C Baldridge, E T Henry, W M Fitch, J D Jordan, E D Yeats '77, S S Shields '77, A H Smith, J A Hanks '77, A P Harris, F M Rushing, Robert Kells, J W Gilbert, Jno A Gordon '77, H Y Hunt '77, T L Darby, J W Patton, B W Doster, Jno Gerdine, O E Newton, M K Harrison, R S Hallsey '77, R S Walker 6mos, Jno Elsner, W J McAlpine, W S Gantier, J W Harris, A A Hill, A E Simpson, C M Gibson, T P Oliver, Robt James, T C Powell, L J Sherrill, J S Knott, J H Knott 6 mos. G R Dozier, W K Chambers, C H Jones, E H M Parham, J W Sanders, Frazer & Wiley, A B McWhorter, J J Grace '77, T S Parham, D B Hamilton, E A Cox, J O Sanders, R J Mathews, P Taylor.

